# Discomfort Evaluation of Truck Ingress/Egress Motions Based on Biomechanical Analysis

**DOI:** 10.3390/s150613568

**Published:** 2015-06-10

**Authors:** Nam-Chul Choi, Sang Hun Lee

**Affiliations:** Intelligent HMI/CAD Lab, Graduate School of Automotive Engineering, Kookmin University, 77 Jeongneung-ro, Seongbuk-gu, Seoul 136-702, Korea; E-Mail: naamus@dayou.co.kr

**Keywords:** discomfort evaluation, ingress/egress, ergonomic design, truck footstep, biomechanics

## Abstract

This paper presents a quantitative discomfort evaluation method based on biomechanical analysis results for human body movement, as well as its application to an assessment of the discomfort for truck ingress and egress. In this study, the motions of a human subject entering and exiting truck cabins with different types, numbers, and heights of footsteps were first measured using an optical motion capture system and load sensors. Next, the maximum voluntary contraction (MVC) ratios of the muscles were calculated through a biomechanical analysis of the musculoskeletal human model for the captured motion. Finally, the objective discomfort was evaluated using the proposed discomfort model based on the MVC ratios. To validate this new discomfort assessment method, human subject experiments were performed to investigate the subjective discomfort levels through a questionnaire for comparison with the objective discomfort levels. The validation results showed that the correlation between the objective and subjective discomforts was significant and could be described by a linear regression model.

## 1. Introduction

### 1.1. Research Background

Recently, automotive manufacturers have had great interest in the development of a human-centered vehicle that can account for the driver’s safety and convenience. In the case of large vehicles, particularly cab-over-engine (COE) trucks, footsteps are positioned high above the ground, in front of the front wheel, and are retracted toward the inside of the vehicle, which causes physical discomfort and a very large risk of falling when the driver enters and exits the cabin [1]. According to statistics from Lin *et al.* [2], slips and falls make up 27% of all truck accidents, and they are rated in second place in terms of severity, just behind motor vehicle accidents. Therefore, in order to enhance the driver’s safety and comfort during truck ingress/egress, it is necessary to design and evaluate the truck steps from the perspective of ergonomics [3,4,5].

Evaluation of the discomfort of vehicle ingress/egress has been performed by human subject experiments with a full-size mockup of several typical steps, followed by the administration of a questionnaire for a subjective evaluation [6]. This method has the advantage of directly surveying user discomfort, but has the drawback of relying on the subjective views of the participants, which makes it difficult to obtain objective and quantitative assessment results to compare design alternatives.

Recently, in order to overcome this limitation, several studies have been performed using quantitative evaluation methods of the discomfort during vehicle ingress/egress [4,6,7,8,9,10,11,12]. In these studies, electromyograms (EMGs) of certain muscles were conducted [7] or kinematic or kinetic simulations of movements were performed, which allowed the discomfort to be calculated on the basis of the joint angles and/or loads [4,8,9,10,11,12,13]. However, because the movements of the human body occur by means of the contraction of muscles, the data obtained from measuring or estimating the contraction activity of the muscles involved would be the most desirable source of information for assessing discomfort [14]. Through car ingress/egress experiments using EMG data, in particular, maximum voluntary contraction (MVC) ratios of the main muscles have been observed to contribute to discomfort [7]. However, EMG data can only be obtained for the muscles near the skin. Therefore, a biomechanical simulation method could be an alternative to estimate the MVC ratios for all muscles in a human body.

This study presents a novel quantitative discomfort assessment method based on the muscle activation forces for ingress/egress movements, which are simulated by biomechanical analysis software using a musculoskeletal model of the human body. More specifically, the method uses the MVC ratio of each muscle activated while entering and exiting a vehicle in order to assess discomfort. To develop the method, a full-size physical mockup of a conventional COE truck cabin with three types of stairways was first constructed for human subject experiments, as well as a human motion capture system. After the recruited participants entered and exited the cabin via the different stairways, a questionnaire was administered to conduct a subjective discomfort evaluation of the stairways. Thereafter, the ingress/egress movement data of a representative participant were collected using the motion capture system, and the ingress/egress movements were simulated using the biomechanical analysis system to calculate the muscle activation forces. Lastly, quantitative discomfort values were calculated for the ingress/egress movements via the stairways using our proposed discomfort assessment model based on the MVC ratio of each muscle. The values were compared with the results of the participants’ subjective discomfort evaluations of the stairways to verify our proposed method.

The contents of the present paper are as follows. Section 1 introduces the related work, as well as the research background and objectives. Section 2 describes the variables and apparatuses for the truck ingress/egress experiment. Section 3 presents the methods by which the truck ingress/egress movements were simulated using commercial biomechanical analysis software. Section 4 introduces a new quantitative discomfort evaluation model based on the biomechanical analysis results and presents the discomfort values calculated using the proposed model. Section 5 describes the participant recruitment methods and the ingress/egress experimental methods performed for each step, after which subjective discomfort values were obtained using surveys. In Section 6, the subjective and quantitative discomforts are compared and discussed. Finally, in Section 7, the conclusions and future work are summarized.

### 1.2. Related Work

In the field of ergonomics, research on the discomfort associated with various postures and movements of the human body have been actively performed to evaluate and improve undesirable workplace conditions or to establish ergonomic guidelines for workplaces [15]. Currently, in particular, studies presenting quantitative criteria for movement discomfort based on a kinetic or kinematic analysis of posture or movement are being actively conducted, and attempts are being made to apply the results to the evaluation of discomfort associated with vehicle ingress/egress movements.

Kee and Karwowski [15] developed ranking systems for the evaluation of joint stress and joint motions based on perceived discomfort measured during an experiment. The experimental results showed that the perceived discomforts were affected by the joint and joint motions. The ranking systems revealed that, while hip and back motions exhibited higher discomfort ratings than any other joint motion, elbow motions were the least stressful of all joint motions. The ranking systems can be used as a valuable guideline when ergonomically designing or evaluating vehicles and workplaces.

Dufour and Wang [8] introduced a novel concept called neutral movement to evaluate the discomfort perceived by humans during vehicle ingress/egress movements and defined the discomfort for each joint and the overall body using the relative discomfort index as the weight of discomfort for each degree of freedom (DOF) of a joint. The proposed discomfort evaluation equation has the advantage of being able to readily calculate the discomfort associated with ingress/egress. However, it is a static discomfort model based on the joint angles and, thus, does not reflect the dynamical aspects of ingress/egress movements.

Debril *et al.* [11] attempted to use a dynamic approach to analyze car ingress/egress motion to overcome this limitation. However, this study has several restrictions, especially because it was limited to a lower-limb analysis of a single subject. For a complex motion, such as car accessibility, which utilizes all body segments, it is necessary to study the entire body.

Causse *et al.* [9] proposed an experimental protocol adapted for analyzing the joint torques and forces of car ingress/egress motion using inverse dynamics. To this end, two preliminary studies based on video analyses were performed to identify the main driver–car interactions and design parameters affecting the driver’s discomfort. Then, an experimental protocol was proposed and applied in a pilot study to verify the feasibility of the protocol. Validation of the calculated joint torques and forces will also be discussed, as well as the relevance of a dynamic approach.

Anderson *et al.* [16] presented a model for calculating the maximum voluntary joint torque as a function of the joint angle and angular velocity. Isometric, concentric, and eccentric maximum voluntary contractions were studied during hip extension, hip flexion, knee extension, knee flexion, ankle plantar flexion, and dorsiflexion. Model parameters are reported for each of these exertion directions by gender and age group. If this model is extended to the joints of the upper body, it can be applied to the discomfort evaluation for vehicle ingress/egress movements.

Namamoto *et al.* [7] presented a method for evaluating the discomfort on the basis of physiological experimental data during vehicle ingress/egress movements. They observed the EMG and MVC ratios of the main muscles contributing to car ingress/egress movements. The main findings are as follows: (1) participants felt major stress when the maximum muscular power ratio exceeded 30% for at least one muscle; (2) in the case in which the representative muscles are the same (same motion pattern and phase), the sensory evaluation of the muscular stress is closely correlated with the sum of the MVC ratios of the representative muscles; and (3) elderly people have a higher muscular stress (approximately 20% higher for people in their 60s). Although this method has a limitation on a full-body discomfort evaluation because EMG data can only be obtained for large muscles near the skin, the idea of a discomfort evaluation based on the MVC ratios is very reasonable. Therefore, we adopted this idea and developed a novel method using the MVC ratios of all muscles in the body obtained through a biomechanical simulation of vehicle ingress/egress movements.

Kim and Lee [17] developed a method to evaluate discomfort while entering into a passenger car to design a side panel for comfortable car ingress. The correlation between the muscle forces and discomfort was investigated, and a discomfort evaluation method based on the muscle forces was developed. To calculate the muscle forces, a biomechanical simulation was performed using the motion data captured by infrared cameras. The mathematical correlation between the calculated muscle forces and the discomfort was obtained by means of fuzzy logic. The main difference between the approach of Kim and Lee and our approach is that we use the MVC ratios, whereas they used the muscle forces themselves. However, the discomfort happens when the actual force approaches the maximum muscle force, and the maximum force varies widely depending on the muscle size. Therefore, the muscle force itself cannot represent the discomfort level correctly. In addition, we applied the discomfort evaluation method to the design of truck steps, whereas they applied their method to the design of the side panel of a passenger car.

The difference between our method and the two previous methods are summarized in Table 1. Our method was developed by combining the advantages of two methods, which were the idea of MVC ratios from Namamoto *et al.* [7] and the estimation method of muscle forces from Kim and Lee [17], and applying this new method to a different area.

**Table 1:** Summary of the previous and current research studies for discomfort evaluation of vehicle ingress/egress motions.

Category		Namamoto *et al.* [7]	Kim and Lee [17]	This Study
Target Area		Car Ingress/Egress	Car Ingress	Truck Ingress/Egress
Independent Variables of Experiments		Height of Hip Point, Height of Floor	Height and Width of Side Panel, Location of A-Pillar	Type And Number of Footsteps, Height of First Step
Parameters for Discomfort Evaluation		MVC Ratio of Muscle Myoelectricity	Muscle Force	MVC Ratio of Muscle Force
Muscle Force Estimation		EMG Test	Biomechanical Analysis	Biomechanical Analysis
(No. of Subjects)	Data Acquisition	10	1	1
	Subjective Evaluation	10	1	10
Muscles Measured or Estimated		13 Muscles	All Muscles	All Muscles
Muscles Used for Discomfort Evaluation		3 Muscles	11 Muscles	All Muscles
Correlation between Muscle Forces and Subjective Discomfort		Linear Regression	Fuzzy Logic	Linear Regression

## 2. Experimental Methods

### 2.1. Experimental Design

#### 2.1.1. Independent Variables

To evaluate the discomfort of ingress/egress movements for COE trucks, we chose three independent variables for the truck stairway, which are the number of steps, height of the first step above the ground, and stairway type. The cab of the TATA DAEWOO Novus heavy truck was selected as a baseline for the experiments. This cab has a floor height of 1360 mm and two 340 mm × 170 mm steps. The heights of the first and second steps are 450 mm and 905 mm, respectively.

**Figure 1:**
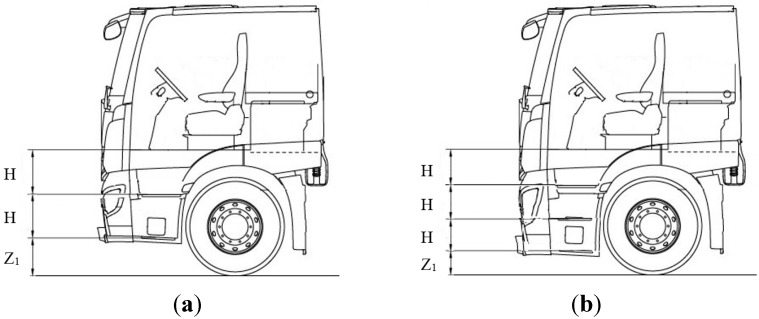
The steps of the truck cab (Z_1_ is the height of the first step, and H is the gap between steps): (**a**) two steps and (**b**) three steps.

As for the number of steps, two or three steps were selected, as illustrated in Figure 1. The height of the first step above ground was chosen as a variable, and it was assumed that the gaps between steps were the same. In the case of a stairway with two steps, the height of the first step was set at 450, 575, or 700 mm, whereas in the case of one with three steps, it was set at 340, 510, or 600 mm.

**Figure 2:**
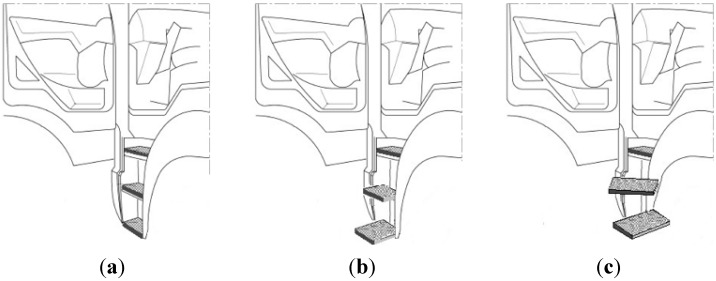
Types of truck steps: (**a**) ladder; (**b**) straight stair; and (**c**) oblique stair.

As for the type of stairway, we selected three different types: ladder, straight stair, and oblique stair. The ladder type, as shown in Figure 2a, is generally used in COE trucks and has two or three steps that retract into the body of the truck. The straight-stair type, as shown in Figure 2b, is a stairway with moving steps that temporarily protrude straight out at the time of ingress/egress, allowing someone to enter and exit the cabin as if ascending or descending stairs. The oblique-stair type, as shown in Figure 2c, has steps that temporarily protrude obliquely-forward and sideways.

#### 2.1.2. Dependent Variable

The discomfort was selected as the dependent variable for the truck ingress/egress movement experiments and simulations. This quantitative variable is presented by a discomfort rating value derived from the results of a questionnaire or the biomechanical analysis of a specific movement. For a subjective evaluation of discomfort in the questionnaire, participants respond with regard to the perceived full-body comfort and a set of selected joints using the nine-point Likert scale from “extremely poor” to “extremely good”. Here, the selected joints include the neck, shoulders, elbows, wrists, lumbar, hips, knees, and ankles. The scores for the nine verbal categories are converted to a percentage scale of the discomfort rating values, as listed in [15].

For an objective measurement of the discomfort, a biomechanical analysis of a musculoskeletal full-body model for ingress/egress movements is performed using a commercial software package, LifeMOD [18], to calculate all joint loads and muscle forces. The discomfort is evaluated using a newly proposed formula based on the MVC ratios of the muscles.

### 2.2. Apparatus

#### 2.2.1. Mockup of the Truck Cabin and Steps

**Figure 3:**
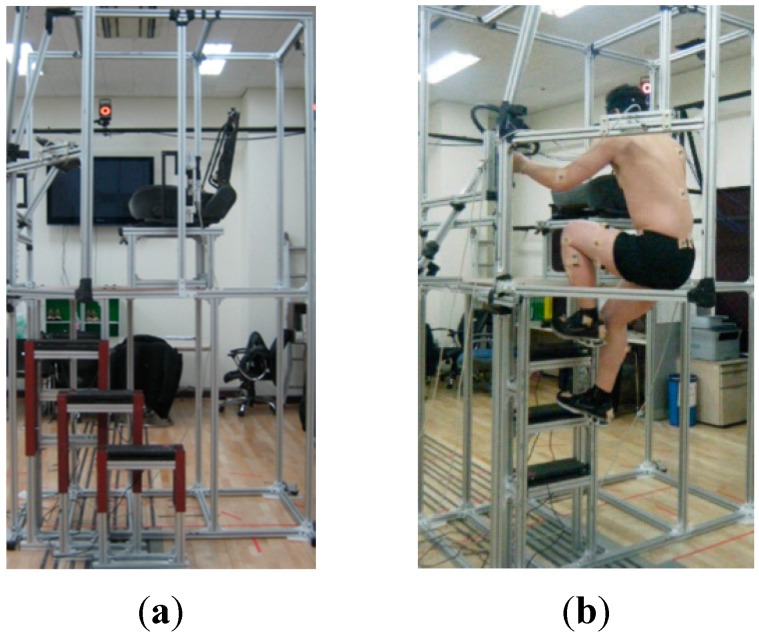
Full-size physical mockup of a truck cabin: (**a**) a cab with portable steps that are configured as straight and oblique stairs and (**b**) a cab with ladder-type steps that are the traditional stairway of COE trucks.

**Figure 4:**
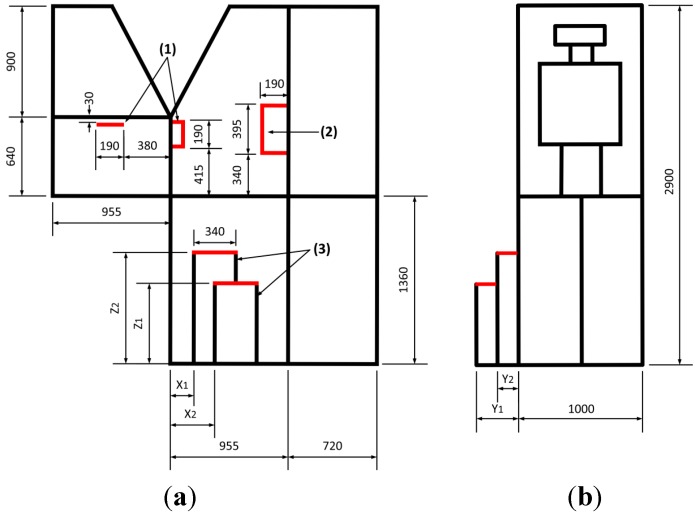
Drawings of the truck mockup (all of the numbers in the subfigures are in millimeters): (**a**) front view and (**b**) side view.

A full-size truck mockup was built for the ingress/egress experiment, as shown in Figure 3. In order to make straight and oblique stairs, three portable, adjustable-height steps were constructed, as shown in Figure 3a. The experimental mockup was built within a minimal frame using aluminum profiles so that body movements could be readily captured by the cameras of an optical motion capture system. In order to prevent reflection noise, the glossy parts were covered with an opaque powder. Figure 4 shows the dimensions of the truck steps. The location and height of a step are denoted by X_i_, Y_i_, and Z_i_. Table 2 lists the locations and heights of the steps for all eighteen cases for the truck ingress/egress experiment.

**Table 2:** All eighteen cases for truck ingress/egress experiments.

No. of Steps	Type of Step	Step Location and Height (mm)									Case No.
		First Step			Second Step			Third Step			
		X_1_	Y_1_	Z_1_	X_2_	Y_2_	Z_2_	X_3_	Y_3_	Z_3_	
2	Ladder	0	0	450	0	0	905	-	-	-	1
		0	0	575	0	0	967	-	-	-	2
		0	0	700	0	0	1030	-	-	-	3
	Straight Stair	0	340	450	0	170	905	-	-	-	4
		0	340	575	0	170	967	-	-	-	5
		0	340	700	0	170	1030	-	-	-	6
	Oblique Stair	340	340	450	170	170	905	-	-	-	7
		340	340	575	170	170	967	-	-	-	8
		340	340	700	170	170	1030	-	-	-	9
3	Ladder	0	0	340	0	0	680	0	0	1020	10
		0	0	510	0	0	793	0	0	1076	11
		0	0	600	0	0	853	0	0	1106	12
	Straight Stair	0	510	340	0	340	680	0	170	1020	13
		0	510	510	0	340	793	0	170	1076	14
		0	510	600	0	340	853	0	170	1106	15
	Oblique Stair	510	510	340	340	340	680	170	170	1020	16
		510	510	510	340	340	793	170	170	1076	17
		510	510	600	340	340	853	170	170	1106	18

#### 2.2.2. Motion Capture System

**Figure 5:**
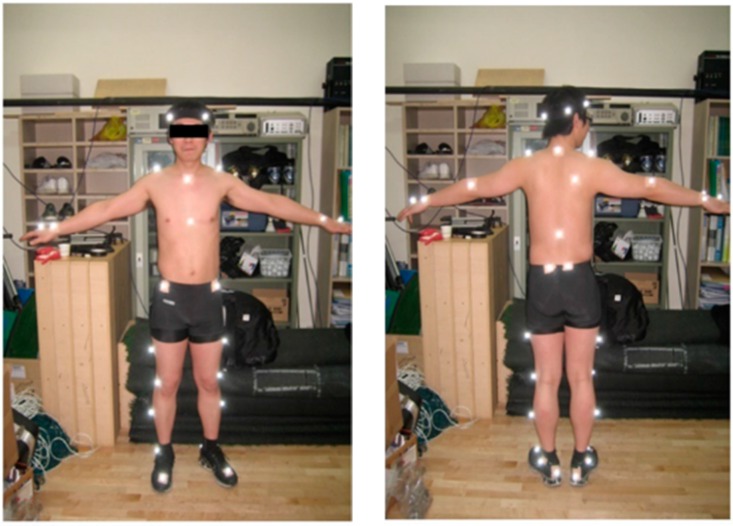
A set of markers is attached to a subject in order to capture ingress/egress movements using the infrared cameras of the Vicon MX system.

In order to capture the truck ingress/egress movements, 10 infrared cameras of a Vicon MX system [19] were installed, and 35 markers were attached to participants, according to the positions of the marker set of LifeMOD, as shown in Figure 5. The truck ingress begins from the moment that a subject, initially in a standing position, lifts one foot from the ground to the moment that he sets both feet on the cabin floor after ascending the stairway. The egress begins from the moment that a subject, initially in a seated position, lifts one foot from the cabin floor to the moment that he sets both feet on the ground after descending the stairway. The motion capture data were converted and exported by Nexus to the .slf data file format that LifeMOD can import as an input motion data file.

#### 2.2.3. Force Measurement Instruments

**Figure 6:**
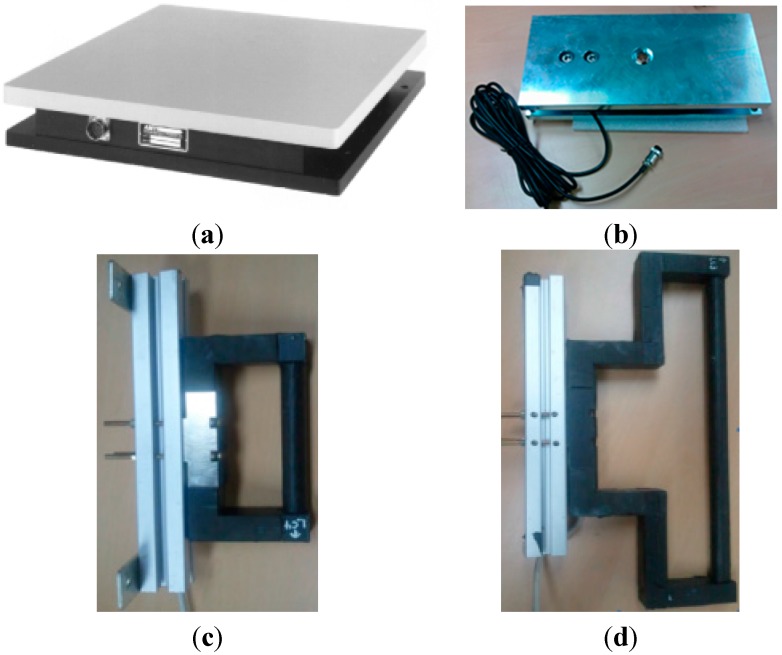
Reaction force measurement devices: (**a**) AMTI OR6-7 force plates on the ground; (**b**) force plate on a step; (**c**) three-axis force handle attached to the A pillar and door marked by (1) in Figure 4a, and (**d**) three-axis force handle attached to the B pillar marked by (2) in Figure 4a.

In order to validate the boundary conditions for biomechanical analysis, eight force measurement devices were prepared. The sampling frequency of all measurement devices was 1 kHz. Two AMTI OR6-7 force plates [20] were placed on the ground to measure the reaction force (max. 450 kgf) against the sole of the foot, as shown in Figure 6a. The measurement device crosstalk is less than 2% on all channels, and the F_x_, F_y_, and F_z_ hysteresis and nonlinearity are ±0.2% of the full-scale output. As shown in Figure 6b, two or three force plates were placed on the steps at location (3) in Figure 4a to measure the reaction force (max. 200 kgf) of the steps against the foot. The error rate of the force plates is 0.03%. In order to measure the three-axis handle reaction force (max. 100 kgf) against the hand, three handles were fabricated based on CurioTec CBSM-100L shear whose combined error is 0.03%, as shown in Figure 6c,d. Two handles, shown in Figure 6c, were attached to the A pillar and the door at location (1) in Figure 4a, and one handle, shown in Figure 6d, was attached to the B pillar at location (2) in Figure 4a.

#### 2.2.4. EMG Measurement Instrument

**Figure 7:**
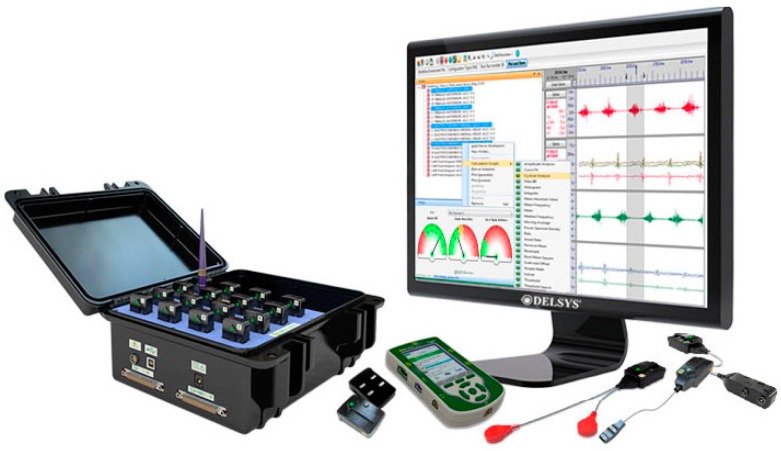
DELSYS Trigno wireless EMG measurement instrument.

In order to measure and record surface EMG activity during the ingress/egress movements, we used the DELSYS Trigno wireless EMG instrument shown in Figure 7 [21], which was fully integrated with the motion capture system. Each EMG sensor has a built-in triaxial accelerometer, a transmission range of 40 m, and a rechargeable battery. In this study, eight wireless EMG sensors were attached to the skin above the muscles of the lower body, which are the hamstrings, biceps femoris, tibialis anterior, and gastrocnemius of both legs. The forces activated by those muscles were simulated by a biomechanical analysis system and then compared with the EMG measurement data to validate the analysis results.

## 3. Biomechanical Analysis

Biomechanical analyses of the truck ingress/egress movements were performed using LifeMOD, which is virtual human modeling and simulation software implemented on the basis of ADAMS human figure modeler [18]. The musculoskeletal model for a subject’s body was created, the markers were attached to the model, and the boundary conditions at the contact positions between the subject and truck were specified. Then, the biomechanical analysis software simulated the muscle forces, as well as the joint angles and loads for the given input data. The analysis results were stored at 1/100 s intervals in this study.

### 3.1. Markers and Motion Data

**Figure 8:**
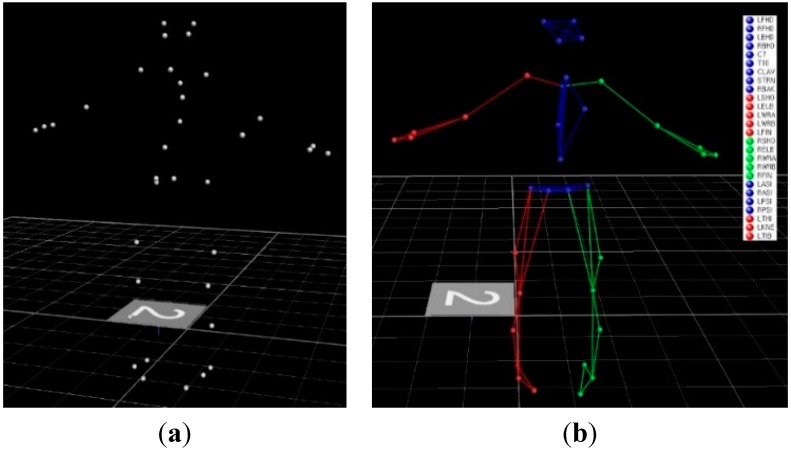
Markers for motion capture: (**a**) creation of a set of motion markers and (**b**) defining the motion markers for LifeMOD.

In order to provide the biomechanical analysis system with the ingress/egress movement data, markers were first defined for the human body model, and the trajectory of each marker was then selected in LifeMOD, as illustrated in Figure 8. In the experiment, 35 markers were attached to the participant to trace their locations at each time interval for the ingress/egress movements. The VICON Nexus utility was used to repair the missing or noisy marker data and to export data files in the .slf format so that the movement data could be analyzed with LifeMOD.

### 3.2. Musculoskeletal Human Body Model

**Figure 9:**
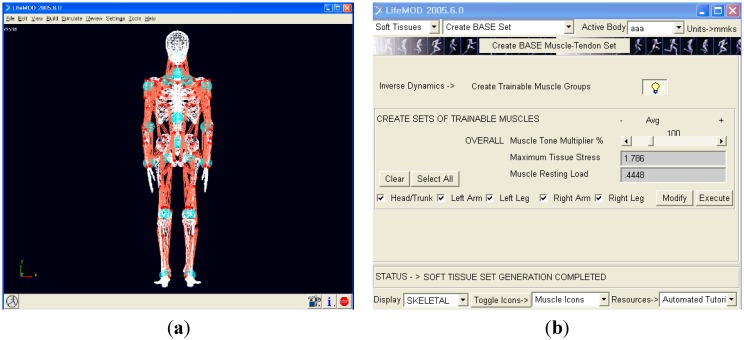
Human body model in LifeMOD: (**a**) musculoskeletal model and (**b**) muscle properties.

The human body model of LifeMOD used in this study consists of 19 segments, 18 joints, and 112 muscles. As shown in Figure 9, the model was configured to match the body dimensions of the participant, and the default LifeMOD values were applied for the material properties of the joints and muscles. The maximum stress on a muscle was 1.786 N/mm^2^, and the maximum contraction force for each muscle was obtained by multiplying the cross-sectional area (ρCSA) of that muscle by the maximum stress.

### 3.3. Boundary Conditions for Biomechanical Analysis

**Figure 10:**
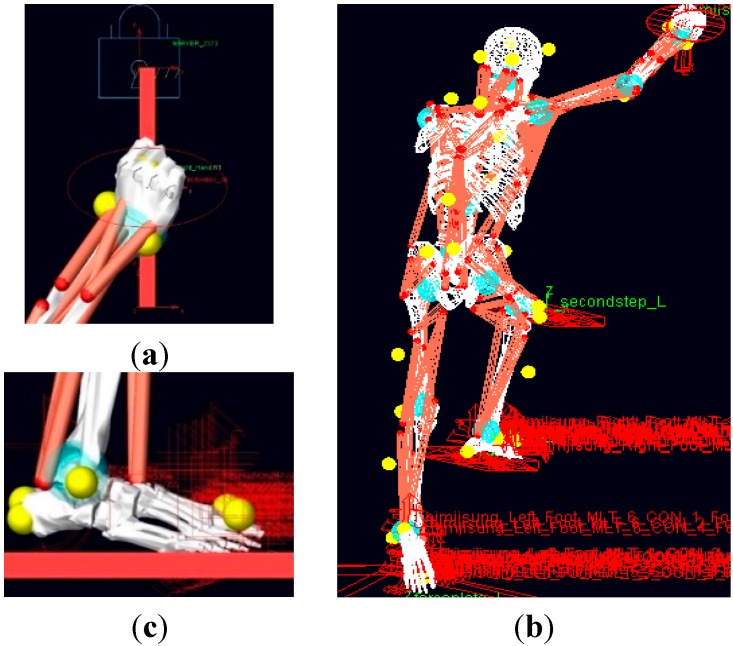
Modeling the boundary conditions at the contact positions between the human body and the environment: (**a**) bushing condition between the hand and the handle; (**b**) contact condition between the foot and the step; and (**c**) boundary conditions applied to the full body.

While entering and exiting a truck cabin, the body is in contact with the steps and handles, creating locations for where the reaction forces occur. These external forces were applied to the simulated body by modeling the boundary conditions appropriately. For this purpose, the contact conditions between the foot and the step and between the hand and the handle were modeled as follows. First, as shown in Figure 10b, contact conditions were created between the foot and the step for the generation of the step reaction forces. For the contact conditions, the contact forces in the surface normal directions were calculated using an impact force algorithm for ellipsoid-plane elements. Here, the impact force is modeled as a function of the penetration depth and velocity and the stiffness and damping coefficients. In addition, the contact forces in the tangential direction were calculated from the velocity-based friction force model, which is a function of the static and dynamic friction coefficients that vary with the slip velocity. The attributes of the contact conditions could be adjusted, and in this study, the default values of LifeMOD were applied [22]. Second, in order to generate the contact force between the hand and the handle, a bushing element was adopted and modeled. For this purpose, as shown in Figure 10a, two markers were generated on the palm and the handle at the contact point, and a bushing element was created between them so that a handle reaction force could be generated. The properties of the bushing were adjusted on the basis of the data captured by the force-measuring device in the actual experiments. Figure 10c shows the boundary conditions applied to the full-body model.

### 3.4. Validation of the Model with Measured Data

**Figure 11:**
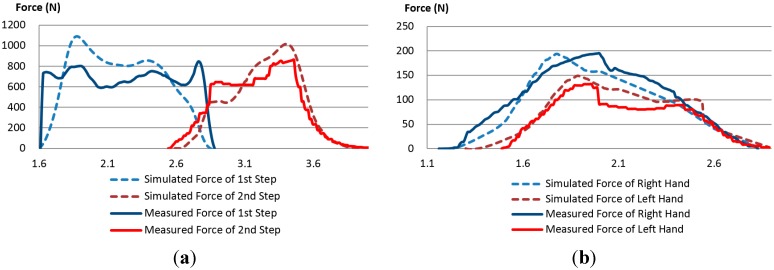
Comparison of the simulated and measured reaction forces of the step and handle: (**a**) reaction forces of the first and second steps and (**b**) reaction forces of the handles on the A and B pillars.

A simulation was performed with LifeMOD using the human body model, boundary conditions, and ingress/egress movement data. As a result, not only the angle, angular velocity, angular acceleration, and torque for each DOF of a joint, but also the contraction force of each muscle during the movement was obtained. In order to validate the boundary conditions for the hand and foot, a comparison was made between the reaction forces of the step and handle measured in the experiment and the external forces simulated by LifeMOD. Figure 11a illustrates the vertical reaction forces of the first and second steps in Case 1 (*i.e.*, ladder type, two steps, 450 mm step height), and Figure 11b shows the reaction forces of the left and right handles during an ingress movement. The dotted line depicts the force simulated by LifeMOD, whereas the solid line depicts the reaction force measured in the experiment. The simulated forces and the actual exerted forces exhibit very similar tendencies and magnitudes, which demonstrates that the boundary conditions applied in the simulation can be considered feasible.

**Figure 12:**
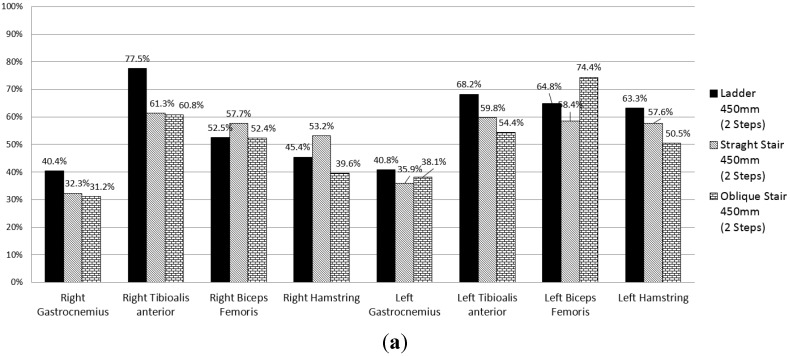

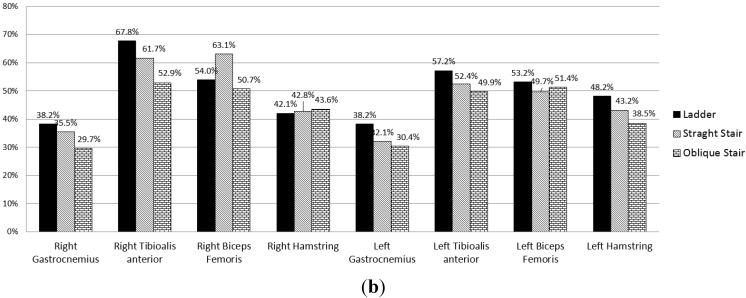
The MVC ratios of the selected muscles during ingress/egress movements: (**a**) MVC ratio calculated on the basis of the EMG data and (**b**) MVC ratio calculated on the basis of the analysis results.

Figure 12a shows the MVC ratios calculated on the basis of the EMG values measured for the hamstring, biceps femoris, tibialis anterior, and gastrocnemius of each leg during ingress/egress movements for three types of stairways consisting of two steps, where the first step has a height of 450 mm. Figure 12b shows the MVC ratios calculated on the basis of the analysis results for the same conditions as in Figure 12a. The error rates of the analysis-based MVC ratio compared with the EMG-based MVC ratio were 11%, 8%, and 12% for the ladder, straight-stair, and oblique-stair types, respectively, with an average error rate of 10%.

## 4. Discomfort Evaluation Based on the Biomechanical Analysis Results

### 4.1. Discomfort Model Based on Muscle Forces

Movement in a human body is caused by the contraction of muscles. The MVC ratio or percent MVC (%MVC) of each muscle during movement can be obtained by the ratio of the actual exerted force (F_i_) to the maximum contraction force (F_i_^max^) that a subject can activate as follows:

(1) $$\gamma_{\text{i}}(t)=F_{\text{i}}(t)/F_{i}^{\max}$$

In this study, the maximum contraction force for each muscle was calculated by multiplying the maximum contraction force per unit area by the cross-sectional area of the muscle from the default material properties of the muscle provided by LifeMOD in order to calculate %MVC. The actual exerted force was obtained from the analysis results of the movement.

The proposed discomfort evaluation formulas based on %MVC for the entire body during the ingress/egress movements are shown in Equations (2) and (3), where m is the number of muscles in the entire body and the start and end of the ingress/egress movements are normalized between zero and one. The discomfort for the entire body at moment *t* is calculated in two ways. First, as described in Equation (2), the highest %MVC is selected as the representative discomfort value for all of the muscles in the body. This is called the maximum-%MVC-based discomfort or simply the maximum-%MVC discomfort.

(2) $$x_{\max}=\int_{0}^{1}\underset{i=1,m}{\max}\left(\gamma_{i}\left(t\right)\right)dt$$

The second way, as described in Equation (3), is by averaging the %MVC values for all muscles and using this value as the representative value of the discomfort. This is called the average-%MVC-based discomfort or simply the average-%MVC discomfort.

(3) $$x_{avg}=\int_{0}^{1}\frac{1}{m}\sum_{i=1}^{m}\gamma_{i}\left(t\right)dt$$

### 4.2. Evaluation of Objective Discomfort Using Discomfort Models Based on the MVC Ratio

In this study, the forces exerted by 112 muscles during truck ingress/egress movements were simulated by LifeMOD. During the ingress movement, the muscles exhibiting high forces were mainly the hips and thighs in the lower body and the back and shoulder muscles in the upper body. The discomfort rating values calculated using the simulated %MVCs of the muscles for each case are summarized in Table 3. The maximum- and average-%MVC discomforts were calculated using Equations (2) and (3), respectively, and their changes, with respect to the discomfort of Case 1 (ladder type, two steps, 450 mm step height), were estimated as well. The case with the least discomfort was Case 7 (oblique stair, three steps, 340 mm step height), and the maximum- and average-%MVC discomforts decreased by 20.5% and 25.7%, respectively.

**Table 3:** Maximum- and average-%MVC discomforts for the eighteen truck step conditions.

No. of Steps	Stair Type	Height of First Step (mm)	Case No.	Maximum-%MVC Discomfort		Average-%MVC Discomfort	
				Value (%)	Change in Rate (%)	Value (%)	Change in Rate (%)
2	Ladder	450	1	60.5	0.0	6.0	0.0
		575	2	64.5	6.6	6.3	4.6
		700	3	67.3	11.3	6.4	6.5
	Straight Stair	450	4	58.4	−3.5	5.8	−3.6
		575	5	62.0	2.5	6.0	0.0
		700	6	67.9	12.2	6.2	2.0
	Oblique Stair	450	7	52.6	−13.2	5.2	−13.6
		575	8	58.3	−3.8	5.8	−3.3
		700	9	61.0	0.8	6.0	−1.5
3	Ladder	340	10	53.0	−12.5	5.7	−5.6
		510	11	55.1	−9.0	5.8	−4.1
		600	12	60.0	−0.9	6.2	1.8
	Straight Stair	340	13	49.9	−17.6	5.1	−15.9
		510	14	54.2	−10.4	5.5	−8.3
		600	15	59.3	−2.1	5.9	−3.1
	Oblique Stair	340	16	48.1	−20.5	4.5	−25.7
		510	17	53.4	−11.8	5.0	−17.6
		600	18	56.1	−7.3	5.4	−11.3

**Figure 13:**
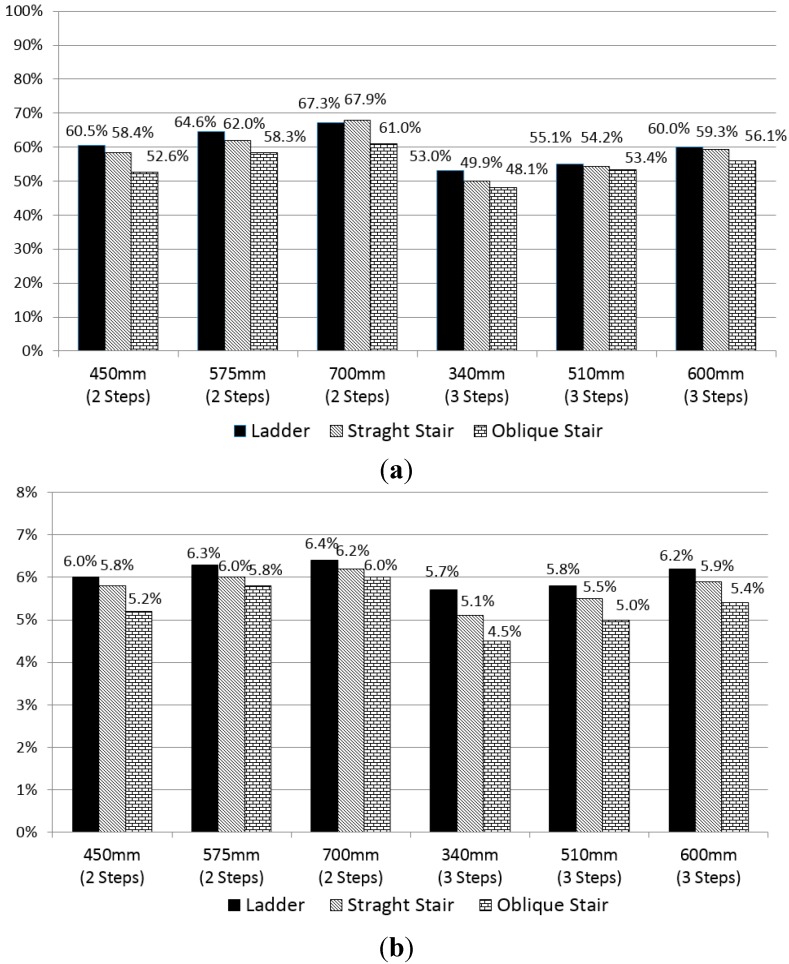
Discomforts for the eighteen truck step conditions according to the step type, step count, and first step height: (**a**) maximum-%MVC discomfort and (**b**) average-%MVC discomfort.

The maximum- and average-%MVC discomforts according to the step type, step count, and step height are illustrated in the bar graphs in Figure 13. The discomfort has a decreasing tendency in the order of ladder, straight stair, and oblique stair. As the number of steps increases, the discomfort decreases, but the discomfort tends to increase as the height of the first step increases. These tendencies agree with the results of the survey of subjective discomfort, as described in Section 5. However, the decreasing rates are somewhat different from those of the subjective discomfort.

## 5. Evaluation of Subjective Discomfort

### 5.1. Participants

A total of 12 undergraduate students (eight males and four females) were enrolled as participants in the experiment. Their average age was 28.3 ± 3.6 years, their average height was 170.3 ± 7.7 cm, and their average weight was 66 ± 12.1 kg. Applicants with a history of musculoskeletal disease, joint impairment, and cognition disorder in the past six months were excluded.

### 5.2. Experimental Procedure

Informed consent was received from participants prior to the beginning of the study. To familiarize themselves with the truck mockup to ensure natural motion during the experiment, the participants practiced entering and exiting the cabin prior to the experiment. Then, the participant performed the ingress/egress for each level of independent variables, and his/her subjective discomfort was surveyed. In the questionnaire, the participants were asked to rate their overall body discomfort during ingress/egress using a nine-point Likert scale from ‘extremely poor’ to ‘extremely good’. In addition, they also rated the discomfort they perceived in their neck, shoulders, elbows, wrists, lumbar, hip joints, thighs, knees, and ankles.

### 5.3. Experimental Results

**Figure 14:**
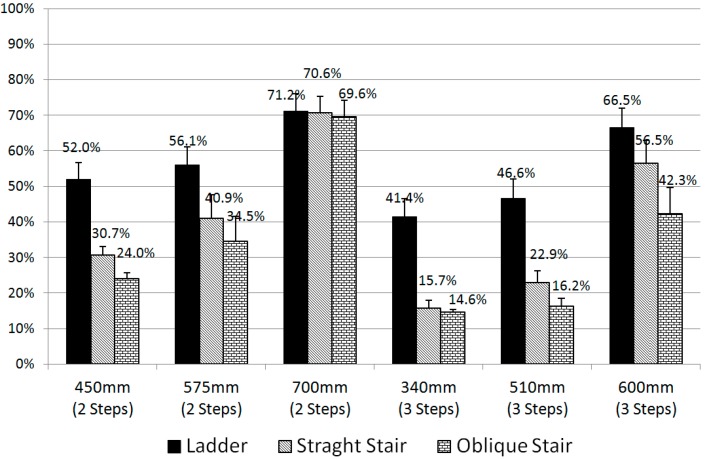
Subjective discomfort of the entire body perceived during truck ingress/egress.

A statistical analysis of the experimental data collected through the questionnaire for subjective discomfort was performed. The verbal terms describing the discomfort on the nine-point scale used in the questionnaire were converted to a percentage scale of perceived discomfort, as listed in Table 4 of Kee *et al.* [15]. The resulting discomfort values for each case are listed in Table 4 and shown in Figure 14. A repeated-measures analysis of variance was conducted using Minitab 16.0 to assess the significance of the three factors: step type, step count, and height of the first step. Significant effects were detected for the step type (*F_2,22_* = 31.41, *p* < 0.001), for the step count, (*F_1,11_* = 29.45, *p* < 0.001), and for the height of the first step (*F_2,22_* = 58.62, *p* < 0.001). The change in the rate of discomfort for a particular case is defined as the percent change from the base discomfort value of Case 1 (ladder, two steps, 340 mm step height) to the case in question. Case 16 (oblique stair, three steps, 340 mm step height) exhibits the minimum discomfort, whereas Case 3 (ladder, two steps, 700 mm step height) exhibits the maximum discomfort, which decreased by 71.9%. The discomfort had a tendency to decrease as the type of stair changed from ladder to straight stair to oblique stair. The discomfort decreased as the number of steps increased from two to three. However, the discomfort increased as the height of the first step increased.

**Table 4:** Survey results of subjective discomfort for the eighteen truck step conditions.

No. of Steps	Stair Type	Height of First Step (mm)	Case No.	Subjective Discomfort		
				Value (%)	Change in Rate (%)	Standard Error (%)
2	Ladder	450	1	52.0	0.0	4.6
		575	2	56.1	7.9	5.0
		700	3	71.2	36.9	4.8
	Straight Stair	450	4	30.7	−41.0	2.4
		575	5	40.9	−21.3	6.8
		700	6	70.6	35.8	4.7
	Oblique Stair	450	7	24.0	−53.8	1.7
		575	8	34.5	−33.8	7.1
		700	9	69.6	33.8	4.6
3	Ladder	340	10	41.4	−20.4	5.1
		510	11	46.6	−10.4	5.5
		600	12	66.5	27.9	5.5
	Straight Stair	340	13	15.7	−69.8	2.2
		510	14	22.9	−56.0	3.4
		600	15	56.5	8.7	6.5
	Oblique Stair	340	16	14.6	−71.9	0.8
		510	17	16.2	−68.8	2.3
		600	18	42.3	−18.7	7.4

## 6. Discussions

### 6.1. Regression Analyses of the Subjective and Objective Discomfort Values

**Figure 15:**
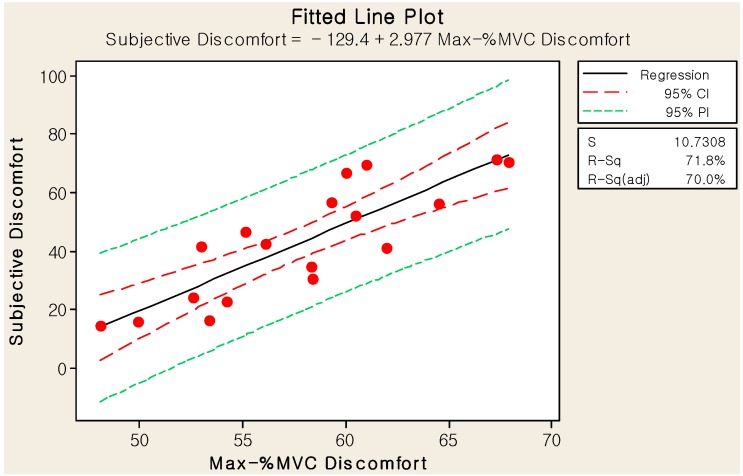
Linear regression model of the maximum-%MVC and subjective discomforts.

We explored the relationship between the objective and subjective discomforts for truck ingress/egress by plotting the two measures against each other for all experimental cases and computing their correlations. The correlation between the maximum-%MVC and subjective discomforts was significant (R = 0.847, *p* < 0.001), such that the large maximum-%MVC-discomfort deviations corresponded with the large subjective discomfort effects in the positive direction. As shown in Figure 15, the linear regression equation was determined to be:

(4) $$\hat{\text{y}}=-129+2.98{\text{ x}}_{\text{max}}$$

where $\hat{\text{y}}$ is the subjective discomfort and x_max_ is the maximum-%MVC discomfort. The standard error of the slope is 0.467 (*t* = 6.38, *p* < 0.001), the standard deviation of the residuals (SE) is 10.73, and the coefficient of determination (R^2^) is 71.8%.

**Figure 16:**
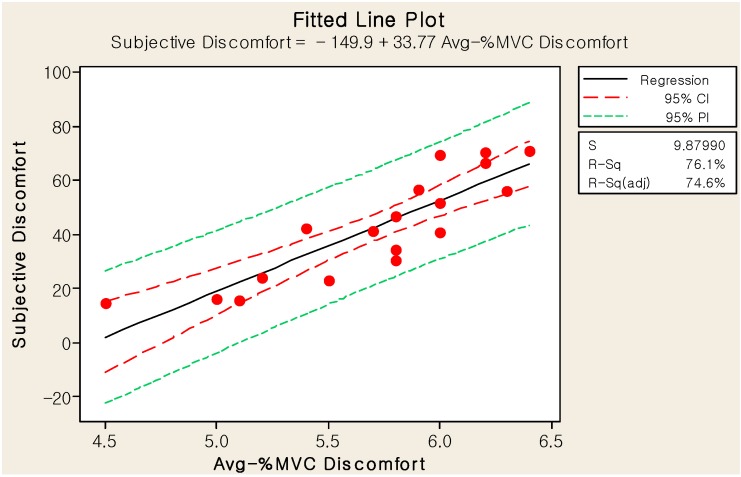
Linear regression model of the average-%MVC and subjective discomforts.

The correlation between the average-%MVC and subjective discomforts was also significant (R = 0.872, *p* < 0.001), such that the large average-%MVC-discomfort deviations corresponded with the large subjective discomfort effects in the positive direction. As illustrated in Figure 16, the linear regression equation was determined to be:

(5) $$\hat{\text{y}}=-150+33.8{\text{ x}}_{\text{avg}}$$

where $\hat{\text{y}}$ is the subjective discomfort and x_avg_ is the average-%MVC discomfort. The standard error of the slope is 4.73 (*t* = 7.13, *p* < 0.001), SE is 9.88, and R^2^ is 76.1%. Therefore, the linear regression model of the average-%MVC discomfort fit the relationship of the objective and subjective discomforts better than that of the maximum-%MVC discomfort when comparing their R^2^ values.

### 6.2. Comparison of the Subjective and Objective Discomforts

The subjective discomfort indicates the perceived body discomfort of the surveyed participants, and the objective discomfort is the maximum- or average-%MVC discomfort calculated from the biomechanical analysis results of ingress/egress movements, as illustrated in Figure 13 and Figure 14. As the number of steps increased from two to three, the subjective discomfort decreased by 28%, the maximum-%MVC discomfort decreased by 11%, and the average-%MVC discomfort decreased by 8%. As the height of the first step increased, the maximum- and average-%MVC discomforts, as well as the subjective discomfort, increased. As for the type of steps, it was shown that the discomfort has a tendency to decrease as the type of steps changes in the following progression: ladder, straight stair, and oblique stair. Compared with the ladder, the subjective discomfort for the straight stair and oblique stair decreased by 31% and 43%, respectively; whereas the maximum-%MVC discomfort decreased by 2% and 8% and the average-%MVC discomfort decreased by 5% and 10%, respectively. The large decreasing rates of the subjective discomfort levels might originate from psychological relaxing effects rather than biomechanical ones.

We also compared the ranking of the eighteen cases for the maximum- and average-%MVC discomforts with that of the subjective discomfort. The root mean square errors of the ranks for the maximum- and average-%MVC discomforts were 12.6 and 10.2, respectively. The averages of the absolute errors of the ranks for the maximum- and average-%MVC discomforts were 2.4 and 1.9, respectively. Therefore, we concluded that the average-%MVC discomfort could predict the subject discomfort more precisely than the maximum-%MVC discomfort. Further, we concluded that the regression equation, Equation (5), could estimate the %MVC discomfort better than Equation (4).

### 6.3. Limitations of the Proposed Method

The objective discomfort evaluation method proposed in this study uses the MVC ratios of the muscle forces calculated by a commercial biomechanical analysis system, LifeMOD, based on a musculoskeletal human body model. However, it is well known that the human body has many more muscles than DOFs of joints and, thus, can have a large number of combinations of muscle forces for a joint torque. Therefore, the accuracy of this method relies on the accuracy of the muscle force calculated by the optimization algorithm used in the movement simulation system. In particular, because the maximum-%MVC discomfort evaluation method selects the highest %MVC value at a certain moment to represent the discomfort of the entire body, a distorted discomfort value could be chosen in the case where an irregularly high %MVC value occurs in a certain muscle.

The decreasing rate of the subjective discomfort is high compared with the decreasing rate of the %MVC discomfort when the type of step is changed from ladder to stair. The reason is thought to be a result of some psychological factor that may contribute to comfort. For example, in the case of the stair-type steps, the user can easily see the steps during egress, and therefore, a dramatic reduction in the risk of an accident, as well as an increase in comfort, is seen. According to many reports, for conventional ladder-type steps, drivers jump from the cabin during egress because the steps are not readily visible, which has led to injuries.

Regarding the generalization of our results to the entire population, including elderly people, it is necessary to enlarge the subject sample size, not only for the subjective discomfort survey, but also for the biomechanical analysis. Currently, the objective of this study is to propose and verify a methodology to rate the subjective discomfort from human ingress/egress motion that may be measured or simulated. The methodology will greatly contribute to the realization of the virtual product design and verification process presented in the next section.

### 6.4. Virtual Product Development Process

The conventional design process, considering the interaction between human and product, adopts the experimental method that uses physical prototypes and human subjects or their dummies. It is both costly and time-consuming to make the prototypes and perform the experiments. Thus, automotive manufacturers strive to reduce product delivery time and cost by replacing the physical prototypes with virtual prototypes and by replacing experiments with computer simulations. If human subjects are replaced with virtual humans, all virtual tests can be performed on a computer. As a result, human-subject related experiments and physical prototypes for ergonomic evaluation will decrease dramatically, and accordingly, the cost and delivery time of the product will be reduced [23,24,25].

**Figure 17:**
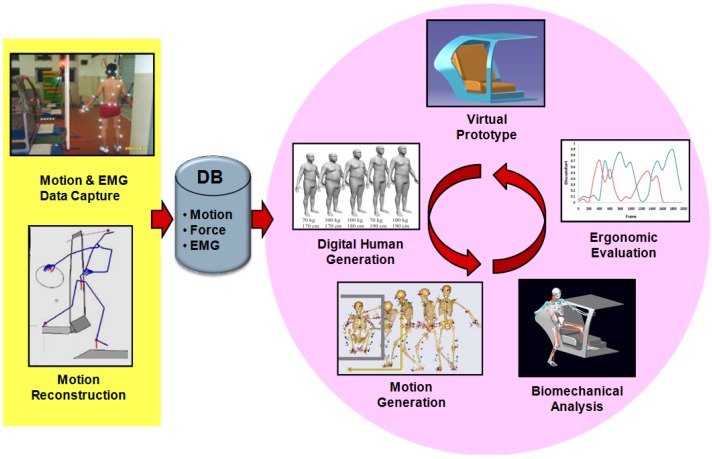
Virtual vehicle design and analysis cycle based on virtual human models and prototypes.

The virtual design and testing process for vehicle ingress/egress proceeds as illustrated in the right side of Figure 17. For the virtual prototype of a newly designed vehicle, virtual human populations are generated, and their ingress/egress motions are generated by using a motion synthesis algorithm. The joint angles, torques, and muscular forces are calculated by biomechanical analysis systems. The discomfort is assessed by the analysis results. Finally, the designer modifies or redesigns the door system on the basis of the discomfort assessment results to improve ingress/egress comfort. The virtual product development process is the ultimate goal of our research. Once such a process is realized, automotive manufacturers may reduce a certain amount of the cost and time required for building physical mockups and performing human-in-the-loop experiments, and thus enforce their competitiveness in the market.

## 7. Conclusions

In this paper, we proposed a new quantitative and objective discomfort evaluation method for truck ingress/egress motions based on muscle MVC ratios calculated by biomechanical analysis. The MVC ratios were calculated for all muscles in the body, and their correlation with the subjective discomfort levels obtained through a questionnaire was modeled using linear regression. The relationship between the %MVC and subjective discomforts was strong (R > 0.84, R^2^ > 71%) and significant (*p* < 0.001). In addition, the linear regression model of the average-%MVC discomfort (R^2^ = 76.1%) fit the relationship between the objective and subjective discomforts better than that of the maximum-%MVC discomfort (R^2^ = 71.8%). This method can be applied to evaluate the discomfort of ingress/egress motions for other types of vehicles, such as cars, heavy machinery, tanks, and combat planes.

For future work, we are developing a virtual ergonomic design and analysis process for vehicles with easy access, which consists of a cycle of design steps, such as the generation of a virtual human model and its motions, a biomechanical analysis, a discomfort evaluation, and, finally, the redesign of products. Here, one of the key steps demanding further study is the synthesis of natural and realistic ingress/egress motions. Prevailing machine learning techniques can be used to solve this problem. The virtual product development process may reduce the cost and time required for building physical mockups and performing human-in-the-loop experiments.
